# Current Perspectives on Mesenchymal Stem Cells as a Potential Treatment for Periodontal Diseases and Conditions

**DOI:** 10.1002/dvg.70024

**Published:** 2025-08-21

**Authors:** Shuyu Cai, Cong Li, Biaowen Wei, Ziyu Ye, Qin Mao, Wanxiang Ye, Mingdeng Rong, Jincheng Zeng

**Affiliations:** ^1^ Department of Stomatology Binhaiwan Central Hospital of Dongguan Dongguan China; ^2^ Department of Stomatology, Dongguan Key Laboratory of Metabolic Immunology and Oral Diseases Dongguan Maternal and Child Health Care Hospital Dongguan China; ^3^ Dongguan Key Laboratory of Medical Bioactive Molecular Developmental and Translational Research, Guangdong Provincial Key Laboratory of Medical Immunology and Molecular Diagnostics Guangdong Medical University Dongguan China; ^4^ Xinghai Institute of Cell Xianhua Medical Research Academy of Guangdong Dongguan China; ^5^ Stomatological Hospital, School of Stomatology Southern Medical University Guangzhou China; ^6^ Department of Stomatology Dongguan Hospital of Integrated Traditional Chinese and Western Medicine Dongguan China

**Keywords:** gingivitis, mesenchymal stem cells, periodontal disease, periodontitis

## Abstract

Periodontal diseases, including periodontitis and gingivitis, constitute a major global health burden, affecting over 1 billion people worldwide. These conditions typically initiate in adulthood and progress chronically, often exhibiting severe manifestations. The socioeconomic impact is particularly acute in low‐ and middle‐income countries, where limited healthcare access exacerbates disease outcomes. Although conventional treatments provide symptomatic relief, they often fail to achieve complete tissue regeneration due to the complex pathophysiology of periodontal destruction. Mesenchymal stem cells (MSCs) have emerged as a transformative therapeutic strategy, demonstrating unique capabilities for immunomodulation, anti‐inflammatory effects, and multipotent differentiation. Preclinical studies have documented MSC‐mediated regeneration of periodontal ligaments, alveolar bone, and cementum through paracrine signaling and direct tissue integration. Clinical trials further substantiate their potential to improve key outcomes, including clinical attachment levels and probing depth reduction. However, five critical challenges require resolution for successful translation: (1) cellular source standardization, (2) mechanistic understanding of long‐term efficacy, (3) safety and immunological profiling, (4) ethical and economic barriers, and (5) clinical translation barriers. This review systematically evaluates current evidence on MSC‐based periodontal regeneration, analyzes these translational challenges, and provides strategic guidance for future research. By integrating fundamental science with clinical perspectives, this work advances the development of reliable MSC therapies for periodontal regeneration.

## 1 Introduction

Periodontal diseases pose a significant threat to overall health due to their high prevalence and severe impacts. Periodontitis and gingivitis, collectively termed periodontal diseases (Worthington et al. 2019), affect the tooth‐supporting structures, leading to progressive tissue destruction and bone loss (Amato et al. 2022). According to 2022 WHO data, severe periodontal diseases affect approximately 19% of adults aged over 15 years globally, representing over 1 billion cases worldwide. Regional prevalence varies significantly: Africa (22.80%), Eastern Mediterranean (17.37%), Europe (17.89%), Americas (18.89%), Southeast Asia (20.77%), and Western Pacific (16.28%). The condition typically emerges around age 55 and persists throughout life, affecting both genders equally with increasing age. Notably, the disease burden has risen substantially in low‐ and middle‐income countries between 1990 and 2019 (World Health 2022).

Periodontal diseases, which initiate as gingivitis (a reversible inflammatory response to dental plaque), can elevate the risk of systemic conditions including cardiovascular disease (Sanz et al. 2020), diabetes mellitus (DM) (Graves et al. 2020), cancer (Nwizu et al. 2020), Alzheimer's disease (Ide et al. 2016), respiratory infections (Herrera et al. 2023), and adverse pregnancy outcomes (Figuero et al. 2020). The transition from gingival health to disease follows a well‐characterized trajectory. Initial plaque accumulation triggers gingivitis, characterized by inflammation confined to the gingiva without attachment loss (Trombelli et al. 2018). Without intervention, this condition progresses to periodontitis, where microbial dysbiosis causes progressive destruction of the periodontal ligament and alveolar bone (Murakami et al. 2018). While current standard treatments effectively manage gingivitis, they remain insufficient for reconstructing lost periodontal tissues, creating a therapeutic niche for MSCs‐based regeneration. Given their capacity for multilineage differentiation and immune modulation, MSCs represent a promising therapeutic strategy for periodontal disease, offering advantages beyond conventional treatments (Amato et al. 2022).

This review systematically evaluates MSCs applications in periodontal regeneration through three specific objectives: (1) elucidating the mechanistic basis of MSCs‐mediated immunomodulation, (2) analyzing preclinical and clinical evidence for tissue regeneration, and (3) identifying key translational challenges and future directions. By integrating current knowledge with emerging research, we establish a critical framework for advancing MSCs therapies from laboratory research to clinical practice in periodontics.

## 2 Periodontal Diseases and Conditions

### 2.1 Periodontitis

The current classification system recognizes three principal forms of periodontitis (Caton et al. 2018). (1) Necrotizing periodontal diseases, which are severe inflammatory conditions characterized by tissue necrosis and frequently associated with systemic immune compromise such as HIV/AIDS or malnutrition (Herrera et al. 2018); (2) Periodontitis, marked by progressive destruction of the periodontal attachment apparatus (Kurgan and Kantarci 2018; Slots 2017), with the 2018 classification introducing staging (to assess disease severity) and grading (to evaluate progression rate) for treatment planning (Papapanou et al. 2018); and (3) Periodontitis as a manifestation of systemic diseases, reflecting the well‐established bidirectional relationship between periodontal and systemic health. This refined classification framework enhances clinical decision‐making through biologically grounded diagnostic criteria.

### 2.2 Other Conditions Affecting the Periodontium

Several additional conditions can compromise periodontal health, including mucogingival deformities that affect tissue morphology and function (Jepsen et al. 2018; Wennström 1996), occlusal trauma caused by abnormal biting forces (Mahendra et al. 2020), and combined endodontic‐periodontal lesions (Foce et al. 2022; Irving 2024). Periodontal abscesses represent acute infectious processes that require prompt intervention (Irving 2024). Importantly, systemic conditions such as diabetes can significantly influence periodontal disease progression and treatment outcomes (Albandar et al. 2018; Jepsen et al. 2018), underscoring the need for comprehensive patient evaluation.

The emerging application of mesenchymal stem cells offers promising potential for addressing these diverse periodontal conditions through regenerative approaches. Accurate diagnosis and effective treatment are essential for maintaining periodontal health and preventing further complications. The emergence of MSCs suggests a new approach for treating periodontal‐related diseases.

## 3 MSCs/DMSCs in the Treatment of Periodontal Diseases

### 3.1 Potential Stem Cells in Dental Regeneration

Stem cells derived from both dental and non‐dental tissues can be used for regeneration. Examples of dental‐derived mesenchymal stem cells (DMSCs) include periodontal ligament stem cells (PDLSCs) (Seo et al. 2004), dental follicle stem cells (DFSCs) (Morsczeck et al. 2005), stem cells from human exfoliated deciduous teeth (SHEDs) (Miura et al. 2003), dental pulp stem cells (DPSCs) (Gronthos et al. 2000), gingiva‐derived mesenchymal stem cells (GMSCs) (Kim et al. 2021), and stem cells from the apical papilla (SCAPs) (Sonoyama et al. 2008). Non‐dental stem cell sources include induced pluripotent stem cells (iPSCs), bone marrow mesenchymal stem cells (BM‐MSCs), adipose‐derived mesenchymal stem cells (AD‐MSCs), and human umbilical cord mesenchymal stem cells (UC‐MSCs) (Shaikh et al. 2022). Table 1 provides a systematic comparison of 6 MSC types (PDLSCs, GMSCs, DPSCs, AD‐MSCs, BM‐MSCs, and UC‐MSCs), evaluating their fundamental biological properties, functional capacities, and clinical potential for periodontal applications. Through comprehensive comparison of these MSC sources across multiple key parameters, ranging from cellular characteristics to therapeutic advantages and limitations, this analysis offers practical guidance for cell source selection in periodontal regenerative strategies. The inclusion of both dental‐derived and non‐dental MSCs highlights the expanding therapeutic toolkit available for personalized periodontal treatment approaches.

**TABLE 1:** Comparative analysis of MSCs types for periodontal regeneration.

MSC type	Characteristics	Harvest procedures	Proliferation ability	Immunomodulatory	Advantages	Limitations
PDLSCs (Malyaran et al. 2024; Qiao et al. 2023)	Spindle‐shaped	Extracted from extracted teeth (e.g., wisdom teeth) by scraping the periodontal ligament. Requires enzymatic digestion (collagenase/dispase).	Moderate to high Slower than GMSCs/DPSCs.	Strong Inhibition of T cell proliferation Modulation of the inflammatory response	Easy to obtain Can differentiate into osteogenic, chondrogenic, and adipogenic lineages.	Affected by periodontal disease Age‐related efficacy declines
GMSCs (Angelopoulos et al. 2018; Dave et al. 2022)	Spindle‐shaped	Collected from gingival tissue during dental procedures Minimal invasiveness (small biopsy) Enzymatic digestion or explant culture.	High Faster doubling time than PDLSCs/BM‐MSCs.	Excellent immunomodulatory function Stronger immunosuppression than DPSC Strong anti‐inflammatory effect	Noninvasive collection Excellent proliferation and angiogenesis Strong immune regulation	Limited tissue volume Relatively low osteogenic capacity
DPSCs (Qiao et al. 2023; Qu et al. 2021)	Spindle‐shaped	Isolated from dental pulp of extracted teeth (e.g., deciduous or permanent teeth) Requires pulp extraction and enzymatic digestion.	High Faster than BM‐MSCs/PDLSCs.	Good immunomodulatory capacity Regulates inflammation Promotes tissue healing	Good osteogenic potential	Tooth extraction required Age affects stemness (better in young donors).
AD‐MSCs (Lau et al. 2024; Lin et al. 2023)	Spindle‐shaped	Obtained via liposuction or fat biopsy (subcutaneous adipose tissue). Collagenase digestion followed by centrifugation.	High Greater expansion capacity than BM‐MSCs.	Strong immunomodulatory effects Inhibition of lymphocyte proliferation Modulation of immune responses	Abundant source Useful for fat, bone, cartilage, and vascular repair.	Collection procedures are invasive Influenced by donor obesity/metabolism Lower osteogenic potential compared to BM‐MSCs/DPSCs.
BM‐MSCs (Meesuk et al. 2022; Wilson et al. 2024)	Spindle‐shaped	Harvested via bone marrow aspiration (iliac crest). Invasive Density gradient centrifugation for isolation.	Moderate Slower doubling time than AD‐MSCs/DPSCs.	Mature immunomodulatory properties Suppression of T‐cell activation Suppression of inflammatory responses	Gold standard of MSCs research Extensive research background Proven clinical efficacy	Invasive collection Age‐related decline in efficacy
UC‐MSCs (Chen et al. 2015; Meesuk et al. 2022; Yi et al. 2020)	Spindle‐shaped	Collected from Wharton's jelly or umbilical cord blood after birth. Non‐invasive source. Enzymatic digestion or explant method.	Strongest Low immunogenicity.	Excellent immunosuppressive properties Strong anti‐inflammatory effects	Non‐invasive collection Strong immunoregulation	Limited by birth events Challenges in standardization Weaker osteogenesis than BM‐MSCs.

### 3.2 Comparison of DMSCs vs. BM‐MSCs vs. UC‐MSCs vs. AD‐MSCs

#### 3.2.1 Regenerative Potential Evaluation

##### 3.2.1.1 DMSCs and BM‐MSCs

The study by Huang et al. systematically compared BM‐MSCs with DMSCs (including SHED, DPSCS, SCAP, PDLSCs, DFPCs) and BM‐MSCs. Their findings demonstrated that both cell types possess self‐renewal capacity and multilineage differentiation potential, giving rise to osteogenic, chondrogenic, adipogenic, myogenic, and neurogenic lineages. Notably, dental stem cells exhibited a marked propensity for odontogenic differentiation, forming dentin‐like structures, whereas BM‐MSCs primarily underwent osteogenic differentiation. The study proposed that the unique differentiation potential of dental stem cells—particularly their enhanced specialization for dental tissue regeneration (e.g., dentin and periodontal ligament), may stem from their neural crest‐associated origin. In contrast, while BM‐MSCs demonstrated broader multipotency for mesenchymal tissues (e.g., bone and cartilage), their capacity to generate tooth‐specific tissues was limited. Consequently, although BM‐MSCs remain widely applicable for general bone regeneration, dental stem cells offer superior potential for tooth and periodontal tissue engineering, especially in the reconstruction of dentin and periodontal ligament structures (Huang et al. 2009; Yang et al. 2010).

##### 3.2.1.2 PDLSCs and UC‐MSCs

A review conducted by Shaikh et al. indicated that both PDLSCs and UC‐MSCs are effective in the treatment of periodontal disease, but each has its own strengths. PDLSCs exhibit a higher osteogenic differentiation capacity, making them particularly efficacious for bone regeneration, while UC‐MSCs have a stronger anti‐inflammatory property, thus more suitable for holistic healing of soft tissue regeneration. Consequently, both cell types were found to be effective, with the choice between the two depending on the specific clinical demands (Shaikh et al. 2022). Previous research by Yu et al. demonstrated that PDLSCs exhibit significantly greater osteogenic differentiation capacity than Wharton's jelly‐derived mesenchymal stem cells (WJMSCs). As osteogenic differentiation is crucial for periodontal tissue regeneration, PDLSCs show superior osteogenic/dentinogenic, adipogenic, and chondrogenic differentiation potential compared to WJMSCs (Yu, Long, et al. 2013). However, another laboratory‐based research by Shang et al. concluded that the difference between the roles of PDLSCs and UC‐MSCs in the regeneration of periodontal tissues was not statistically significant, indicating that UC‐MSCs have comparable effects in periodontal tissue regeneration and can be used as a potential source for this purpose (Shang et al. 2017).

##### 3.2.1.3 PDLSCs and BM‐MSCs

Although PDLSCs are derived from extracted teeth or periodontal tissues and appear readily accessible, their procurement is more challenging in elderly patients due to degenerative periodontal tissue and limited PDLSC availability. In contrast, BM‐MSCs can be obtained through bone marrow aspiration, with well‐established harvesting and expansion techniques, offering stable sources and high controllability. BM‐MSCs have been widely applied in various clinical disorders. An experiment conducted by Du et al. demonstrated that BM‐MSCs promote periodontal tissue regeneration by repairing tissue defects and suppressing inflammatory responses. The study revealed that BM‐MSCs could differentiate into multiple cell types, including periodontal ligament cells, odontoblasts, and alveolar bone cells, significantly improving alveolar bone regeneration rates. Furthermore, BM‐MSCs reduced the expression of inflammatory factors such as TNF‐α, IFN‐γ, and IL‐1β, exhibiting anti‐inflammatory effects.

Compared with PDLSCs, BM‐MSCs present more convenient sourcing and lower risks of immune rejection, making them particularly suitable for elderly patients or clinical settings with limited resources. Although BM‐MSCs injection for periodontitis treatment does not completely restore periodontal tissues to healthy conditions, their easier accessibility and simpler handling procedures give them broader clinical application prospects than PDLSCs. Therefore, BM‐MSCs demonstrate promising regenerative potential and feasibility as a therapeutic approach for periodontal diseases (Du et al. 2014).

#### 3.2.2 Immunomodulatory Properties Analysis

##### 3.2.2.1 PDLSCs and Other MSCs

From the perspectives of immunoregulation and immunosuppression, we will contrast the effects of PDLSCs with those of other MSCs sources in relation to periodontal diseases. To evaluate the potential of PDLSCs for regenerative engineering of periodontal tissues, Wada et al. compared PDLSCs, BM‐MSCs, DPSCs, and gingival fibroblasts (GFs). Their findings indicate that the immunomodulatory activity of PDLSCs is primarily mediated through the inhibition of activated peripheral blood mononuclear cell (PBMC) proliferation, which is critical for addressing periodontal diseases. This inhibitory effect is mediated by soluble molecules such as TGF‐β1, hepatocyte growth factor (HGF), and indoleamine 2,3‐dioxygenase (IDO). Notably, the expression of these factors—particularly IDO—is partially induced by interferon‐gamma (IFN‐γ) released from activated PBMCs, leading to an overall immunosuppressive effect. PDLSCs may serve as a beneficial agent for the treatment of periodontal diseases, as they operate through a non‐cell‐contact dependent mechanism, suggesting their ability to modulate the immune response while aiding in the regeneration of periodontal tissues by reducing inflammation and promoting tissue repair (Wada et al. 2009).

Subsequently, Nuñez et al. further confirmed that the immunosuppressive properties of PDLSCs mainly modulate the immune response by suppressing T‐cell activation and proliferation through the production of prostaglandin E2 (PGE 2) as well as soluble factors. In addition, PDLSCs are less immunogenic since they do not express human leukocyte antigen (HLA)‐II DR and costimulatory molecules, thus reducing the likelihood of immune rejection. These properties contribute to an anti‐inflammatory environment, which is crucial for healing periodontal disease and tissue regeneration (Nuñez et al. 2019). Several specific growth factors, such as vascular endothelial growth factor (VEGF) (Zhang, Shuai, et al. 2020), platelet‐derived growth factor (PDGF) (Nevins et al. 2013), bone morphogenetic proteins (BMP‐2—Wikesjö et al. 2005; BMP‐7—van den Bergh et al. 2000; and BMP‐12—Wikesjö et al. 2004), transforming growth factor‐β (TGF‐β) (Tatakis et al. 2000), and insulin‐like growth factor (IGF) (Chen et al. 2006), enhance periodontal tissue regeneration by promoting the formation of periodontal ligament, cementum, and alveolar bone. These growth factors act in a concentration‐ and time‐dependent manner, modulating cellular responses through signaling pathways. They also regulate immune function, affect gene expression, facilitate the production of cementum, and ultimately contribute to the restoration of the tooth‐supporting appliance (Chen et al. 2009).

##### 3.2.2.2 PDLSCs and AD‐MSCs

As a result of Mohammed's research, AD‐MSCs and their exosomes have been demonstrated to promote periodontal regeneration in conjunction with scaling and root planning (SRP). Both AD‐MSCs and their exosomes facilitate the formation of new and well‐organized periodontal tissues, with the exosome group exhibiting superior outcomes, characterized by reduced inflammation and enhanced tissue generation. Furthermore, the exosomes replicate the healing effects of AD‐MSCs while offering advantages such as easier preparation and greater safety. Collectively, these findings indicate that AD‐MSCs and their exosomes hold significant promise for enhancing the efficacy of non‐surgical periodontal treatments (Mohammed et al. 2018).

After that, an experiment by Li et al. in 2022 further illustrated that AD‐MSCs possess stronger immunoregulatory capabilities, particularly in modulating macrophage polarization toward the anti‐inflammatory M2 phenotype, thereby effectively suppressing inflammatory responses. This process significantly reduces the secretion of pro‐inflammatory cytokines such as TNF‐α and IL‐1β while upregulating M2 macrophage markers (e.g., CD206), further promoting bone regeneration. In contrast, although PDLSCs play a crucial role in periodontal repair, their immunomodulatory capacity is comparatively weaker. Nevertheless, research has revealed that AD‐MSCs enhance the osteogenic potential of PDLSCs under inflammatory conditions by regulating macrophages via the indoleamine 2,3‐dioxygenase (IDO) pathway. Specifically, AD‐MSCs significantly increase the expression of M2 macrophage markers (CD206, CD163) while decreasing M1 markers (TNF‐α, IL‐1β, iNOS). Conditioned medium (CM) from macrophages co‐cultured with AD‐MSCs exerts a protective effect on the osteogenic differentiation of PDLSCs, particularly in inflammatory environments, suggesting that AD‐MSCs not only suppress inflammation but also enhance the osteogenic potential of PDLSCs through macrophage polarization. Furthermore, the immunomodulatory effects of AD‐MSCs are mediated by the IDO‐dependent kynurenine (Kyn)‐AhR‐NRF2 signaling pathway, which plays a critical role in periodontal tissue repair. These findings provide novel therapeutic insights for periodontal regeneration, particularly by leveraging the immunomodulatory potential of AD‐MSCs to reshape the inflammatory microenvironment (Li et al. 2022).

##### 3.2.2.3 GMSCs, aBM‐MSCs and PDLSCs

Shang et al. compared various DMSCs based on their immunomodulatory and anti‐inflammatory abilities to examine their effectiveness in treating periodontal diseases (Shang et al. 2021). GMSCs, alveolar bone mesenchymal stem cells (aBM‐MSCs), and PDLSCs all have unique immunomodulatory properties. GMSCs exhibit strong immunomodulatory and anti‐inflammatory effects, especially in inhibiting the proliferation of PBMCs and the differentiation of T cells into Th1/Th2/Th17 cells. They also affect macrophage polarization toward an anti‐inflammatory phenotype, characterized by increased IL‐10 secretion and decreased TNF‐α secretion. PDLSCs demonstrate low immunogenicity and robust immunomodulatory capacity, primarily through the upregulation of IDO‐1 (which degrades L‐tryptophan and inhibits local immune cell response). They also efficiently suppress T‐cell effector cells and promote Treg cell proliferation. Although aBM‐MSCs have shown immunomodulatory potential, investigations on their specific actions are not as comprehensive as those of GMSCs and PDLSCs. Each of these MSC types contributes to immunomodulation through unique pathways, rendering them valuable in the development of regenerative therapies and disease treatments.

The immunomodulatory and regenerative properties of MSCs make them an attractive option for enhancing the outcomes of periodontal treatments. By reducing inflammation and supporting the regeneration of damaged tissues, MSCs can not only halt the progression of periodontal disease but also reverse some of the damage caused by the disease (Song et al. 2020).

### 3.3 DMSCs in the Treatment of Periodontal Diseases

**FIGURE 1:**
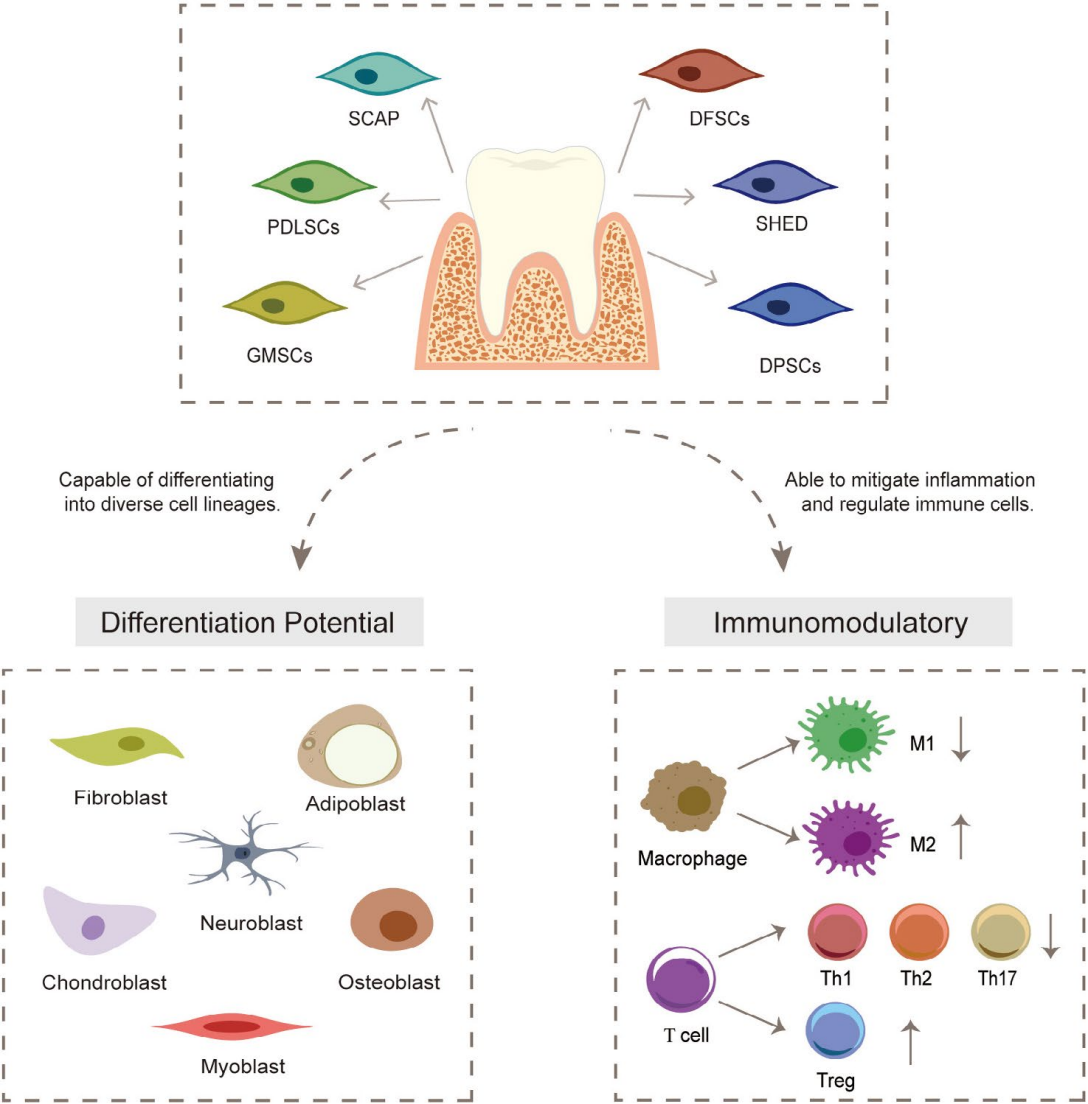
Mechanisms of dental mesenchymal stem cells (DMSCs) in periodontal regeneration, illustrating both differentiation potential (left) and immunomodulatory capacity (right). Abbreviations: DFSCs, dental follicle stem cells; DPSCs, dental pulp stem cells; GMSCs, gingival mesenchymal stem cells; PDLSCs, periodontal ligament stem cells; SCAP, stem cells from apical papilla; SHED, stem cells from human exfoliated deciduous teeth.

The therapeutic potential of DMSCs in periodontal therapy is characterized by their consistent ability to mediate immunomodulation and differentiation potential through fundamentally similar biological pathways. As summarized in Figure 1, the six principal DMSC types (PDLSCs, DPSCs, SHEDs, SCAPs, DFSCs, and GMSCs) engage two distinct functional mechanisms: (1) multilineage differentiation into adipocytes, osteoblasts, fibroblasts, and other cell types; (2) immunoregulation of macrophage polarization states and T‐cell subsets, with therapeutic outcomes being microenvironment‐dependent. This section primarily analyzes the functionality of PDLSCs, GMSCs, and DPSCs, demonstrating their shared regenerative mechanisms while highlighting differences in therapeutic outcomes across experimental conditions. These comparative studies position PDLSCs as the most extensively researched MSCs population for periodontal regeneration applications (Section 3.3.1).

#### 3.3.1 PDLSCs in Preclinical Studies: Regeneration of Cementum, Alveolar Bone, and Periodontal Ligament

PDLSCs demonstrate dual therapeutic potential for periodontal regeneration through their differentiation capacity and immunomodulatory properties. Primarily, PDLSCs can differentiate into cementoblasts, osteoblasts, and fibroblasts, which are essential for reconstructing periodontal tissues including the ligament, cementum, and alveolar bone (Queiroz et al. 2021). Preclinical and clinical studies have confirmed their regenerative efficacy, with measurable improvements in alveolar bone height and clinical attachment levels (Chen et al. 2016; Queiroz et al. 2021; Tassi et al. 2017). Recent advances highlight the critical role of PDLSCs' immunomodulatory function in periodontal therapy. While healthy‐derived PDLSCs (PDLSC‐HPs) effectively regulate inflammation and promote tissue repair, those from inflamed tissues (PDLSC‐IPs) exhibit significant functional impairments. Specifically, PDLSC‐IPs fail to properly control immune responses, leading to T‐helper cell imbalance and excessive T‐cell proliferation, which exacerbates inflammatory bone loss and disease progression.

Notably, pharmacological interventions such as quercetin show promise in restoring the functionality of PDLSCs. Yang et al. (Yang et al. 2024) demonstrated that natural compounds including quercetin can restore the osteogenic and angiogenic capacities of PDLSCs while suppressing inflammatory factors such as IL‐6 and TNF‐α. By modulating macrophage M1 polarization, quercetin improves the local immune microenvironment and indirectly enhances PDLSC function. Specifically, miR‐21a‐5p plays a pivotal role in this process by targeting programmed cell death 4 (PDCD4) and regulating the NF‐κB signaling pathway, thereby coordinately controlling immune responses and amplifying quercetin's immunomodulatory and regenerative effects. This study highlights that reshaping the osteoimmune microenvironment through quercetin represents a promising strategy for achieving in situ alveolar bone regeneration, offering novel therapeutic approaches for periodontitis.

However, most of the existing studies are limited to in vitro experiments, and there is still a lack of in‐depth analysis of its immunomodulatory effects in vivo. With the development of cell lineage tracing technology and gene editing technology (e.g., Gli1, LepR, Axin2), future research can pay more attention to the immunomodulatory mechanism of PDLSCs in vivo, which will help to overcome the challenge of immune rejection in the process of allogeneic transplantation and provide more reliable theoretical support for clinical application (Men et al. 2020; Wang et al. 2022; Zhang, Zhang, et al. 2020). Therefore, in‐depth exploration of the specific molecular mechanism of PDLSCs in immune regulation, especially how PDLSCs regulate immune response in an inflammatory environment, will be an important direction for the development of this field (Wen et al. 2024).

#### 3.3.2 Preclinical Studies on GMSCs: Anti‐Inflammatory Cytokine Secretion and Macrophage Polarization for Periodontal Repair

Previous studies have validated that GMSCs can modulate immune responses by secreting regulatory cytokines and engaging in direct cell–cell interactions. This immunomodulation involves inhibiting T cell proliferation, inducing regulatory T cells, and converting macrophages and dendritic cells into an anti‐inflammatory phenotype (Racz et al. 2014). Additionally, research has concluded that GMSCs may treat periodontal diseases through their immunomodulatory properties by regulating the local inflammatory response (Fawzy El‐Sayed and Dörfer 2016). These cells can suppress pro‐inflammatory cytokines such as TNF‐α, IL‐1β, and IL‐6 (Okić et al. 2021), while facilitating the polarization of macrophages toward an anti‐inflammatory M2 phenotype (Lee et al. 2015). This process leads to elevated levels of anti‐inflammatory cytokines (e.g., IL‐10) and decreased expression of inflammatory cytokines (e.g., IL‐6) (Zhang et al. 2010). Moreover, GMSCs can further alleviate inflammation by blocking the neo‐synthesis of pro‐inflammatory cytokines through the PGE2 mechanism (Zhang et al. 2009; Zhang et al. 2012). Collectively, these actions contribute to reduced local inflammation, enhanced tissue repair, and improved periodontal tissue regeneration, thereby establishing GMSCs as a promising tool for the treatment of periodontal diseases.

#### 3.3.3 DPSCs in Human Studies: Periodontal Tissue Regeneration and Anti‐Inflammatory Effects

DPSCs have yielded promising results in human studies. Research conducted by Monjaraz et al. on elderly individuals demonstrated that DPSCs can differentiate into essential cell types, such as osteoid cells, and possess the ability to generate cementum‐like material from periodontal tissues, thereby facilitating the restoration of periodontal structures. Furthermore, DPSCs exhibit anti‐inflammatory properties, reducing oxidative stress and lowering levels of pro‐inflammatory interleukins, such as IL‐1β. Additionally, DPSCs have shown the potential to secrete cytokines, promote tissue repair, and modulate the immune response, thereby supporting the healing process in periodontal disease. DPSCs can also stimulate the formation of bone‐like tissues, enhance bone mineral density, and promote the proliferation of gingival and osteoblastic cells, which ultimately improves periodontal adhesion and minimizes defects (Hernández‐Monjaraz et al. 2020).

## 4 Preclinical and Clinical Translation of MSC‐Based Periodontal Therapy

### 4.1 Preclinical Evidence Analysis

The analysis of preclinical studies reveals distinct patterns in MSCs applications for periodontal regeneration. For example, in a study using a canine model of periodontitis, MSCs were shown to promote substantial regeneration of alveolar bone, periodontal ligament, and cementum (Rezaei et al. 2019; Sone et al. 2022). Among the 37 studies, dental‐derived MSCs (PDLSCs, DPSCs, GMSCs, and SHEDs) were the most frequently investigated (17 studies, 45.9%), reflecting their tissue‐specific advantages for periodontal repair, while BM‐MSCs (14 studies, 37.8%) were primarily valued for osteogenic potential. Large animal models, particularly beagle/mongrel dogs (13 studies), dominated functional validation studies, whereas rodent models (11 studies) focused on mechanistic insights. Regarding the human‐derived MSC studies, this term encompasses both preclinical researches involving implantation of human tissue‐derived MSCs (from bone marrow or dental pulp) into immunodeficient animal models such as mice, as well as clinical studies transplanting these cells into human patients. The observed heterogeneity in clinical trial designs across those 10 studies primarily stemmed from variations in methodology (ranging from randomized controlled studies (RCTs) to case reports), sample size limitations (particularly in the case–control study), variability in outcome assessment methods (with some studies lacking quantitative bone formation measurements), and inconsistent follow‐up durations (where retrospective designs often lacked long‐term efficacy data). The predominance of ≤ 90‐day evaluations (27 studies) underscores the need for longer‐term durability assessments (Table 2). Future studies should prioritize clinically relevant models integrating chronic inflammation and comorbidities.

**TABLE 2:** Selective studies involving MSCs in the treatment of periodontal diseases (*n* = 37).

Author (year)	Species	MSCs type	Periodontal diseases	Duration (days)
Wei et al. 2010	Beagle dogs	BM‐MSCs	Class III furcation defects	42
Feng et al. 2010	Human	PDLSCs	Periodontitis	2160
Yang et al. 2010	SD rats	Allogenous BM‐MSCs	Periodontal fenestration defects	21
Suaid et al. 2011	Beagle dogs	PDLSCs	Class III furcation defects	84
Park et al. 2011	Beagle dogs	Autogenous PDLSCs, DPSCs	3‐mm‐wide circumferential defect	56
Tsumanuma et al. 2011	Beagle dogs	Autogenous BM‐MSCs, PDLSCs	1‐wall intrabony defects	56
Fawzy El‐Sayed et al. 2012	Minipigs	Autogenous Gingival margin‐MSCs (GM‐MSCs)	Periodontal fenestration defects	84
Nuñez et al. 2012	Beagle dogs	Autogenous PDLSCs	Intrabony defects	84
Simsek et al. 2012	Mongrel dogs	Autogenous BM‐MSCs	Class III furcation defects	56
Suaid et al. 2012	Beagle dogs	Autogenous PDLSCs	Class III furcation defects	84
Zhou and Mei 2012	Beagle dogs	Autogenous BM‐MSCs	Fenestration defects	42
Khorsand et al. 2013	Mongrel dogs	Autogenous DPSCs	Periodontal defects	56
Rosen 2013	Human	BM‐MSCs	Class III furcation defects	180
Koo et al. 2012	Human	BM‐MSCs	Severe periodontitis	270
Yamada et al. 2013	Human	BM‐MSCs	Alveolar bone defects	1800
Du et al. 2014	Rats	BM‐MSCs	Periodontal defects	84
X. Yu, Ge, et al. 2013	Beagle dogs	GMSCs	Furcation defects	56
Mrozik et al. 2013	Merino sheep	Allogenous PDLSCs	Dehiscence periodontal defects	28
Tobita et al. 2013	Beagle dogs	AD‐MSCs and platelet‐rich plasma (PRP)	Class III periodontal tissue defects	60
Han et al. 2014	SD rats	Allogenous PDLSCs	Periodontal fenestration defects	28
Iwasaki et al. 2014	Nude rats	Xenogenous PDLSCs	Class III furcation defects	28
Cai et al. 2015	Fisher rats	Allogenous BM‐MSCs	3‐wall intrabony defects	42
Nagahara et al. 2015	Beagle dogs	Autogenous BM‐MSCs	Class III furcation defects	56
Paknejad et al. 2015	Mongrel dogs	Autogenous BM‐MSCs	3‐wall intrabony defects	56
Wu et al. 2016	SD rats	ADSCs and Amniotic membrane	Periodontal 2‐wall osseous defect	21
Li et al. 2016	Human	DPSCs	Periodontal intrabony defects	270
Chen et al. 2016	Human	PDLSCs	Periodontal intrabony defects	360
Baba et al. 2016	Human	BM‐MSCs	Chronic periodontitis	1080
Mohammed et al. 2018	Rats	AD‐MSCs and their exosomes	Periodontitis	28
Ferrarotti et al. 2018	Human	DPSCs	Chronic periodontitis	360
Gao et al. 2018	Mice	SHEDs	Periodontitis	28
Hernández‐Monjaraz et al. 2020	Human	DPSCs	Periodontal disease	180
Sun et al. 2020	Rabbits	HUC‐MSCs and bone collagen particles	Alveolar cleft bone defects	90
Wofford et al. 2020	SD rats	AD‐MSCs	Maxillary alveolar tooth defects	84
Apatzidou et al. 2021	Human	aBM‐MSCs	Periodontal intrabony defect	180
Li et al. 2022	SD rats	AD‐MSCs	Periodontitis	21
Deng et al. 2024	Mice	Gli1^+^ MSCs	Tooth socket defect	21

*Note:* Key findings: (1) Dental‐derived MSCs (PDLSCs/DPSCs/GMSCs/SHEDs) represented 45.9% (17/37) of studies; (2) Beagle/mongrel dogs constituted the predominant large animal model (13 studies); (3) 73% (27/37) of studies were limited to ≤ 90‐day evaluation periods.

### 4.2 Clinical Trial Evaluation

The data were systematically gathered from the ClinicalTrials.gov database (a global clinical trial registry based in the United States), with a search cutoff date of July 2025 for trials utilizing MSCs for periodontal diseases. The clinical development of MSC therapies for periodontal applications shows steady progress while maintaining a cautious approach. Current clinical trials are primarily in early developmental phases, with Phase 1/2 studies accounting for 12 out of 17 registered trials (70.6%). Notably, 16 of the 17 registered trials (94.1%) enrolled fewer than 50 participants, highlighting the early‐phase nature of most MSCs‐based periodontal therapies. While such small sample sizes are typical for phase 1/2 safety and feasibility studies, they may limit the statistical power to detect clinical efficacy. Geographic distribution showed balanced representation between Asia (8/17, 47.1%) and Europe (6/17, 35.3%), with transcontinental trials (Turkey, Russia) proportionally allocated to both regions. The Americas contributed 23.5% (4/17: 1 North America,3 South America). Notably, no trials originated from Africa or Oceania despite their high periodontal disease burdens (Table 3).

**TABLE 3:** MSCs‐based clinical trials for the periodontal‐related diseases (*n* = 17).

ClinicalTrials.gov Identifier	Phase(S)/Status/(Start Dates)	Enrollment	Conditions	Intervention	Country
NCT06764004	Phase 1 Phase 2 Not yet recruiting (January 2025)	45	Apical periodontitis	UC‐MSCs	Turkey (Europe and Asia)
NCT04446897	Phase 1 Phase 2 Completed (August 2020)	10	Periodontitis	MSCs	Belarus (Europe)
NCT04434794	Phase 1 Phase 2 Completed (January 2018)	27	Gingival recession	MSCs	Belarus (Europe)
NCT06388447	Phase 4 Enrolling by invitation (December 2023)	20	Gingival recession	UC‐MSCs	Malaysia (Asia)
NCT03570333	Not Applicable Active, not recruiting. (September 2018)	7	Gingival recession, Lack of keratinized gingiva	GMSCs	United States (North America)
NCT05924373	Phase 2 Recruiting (November 2023)	204	Periodontitis	DPSCs	China (Asia)
NCT00221130	Phase 1 Phase 2 Completed (July 2004)	10	Adult periodontitis	MSCs	Japan (Asia)
NCT03137979	Phase 1 Phase 2 Unknown status (January 2017)	30	Periodontitis	GMSCs	China (Asia)
NCT03102879	Not Applicable Completed (September 2016)	36	Periapical periodontal	UC‐MSCs	Chile (South America)
NCT02449005	Phase 1 Phase 2 Completed (March 2014)	30	Chronic periodontitis	BM‐MSCs	Greece (Europe)
NCT05599087	Phase 1 Recruiting (December 2022)	10	Periapical periodontitis	UC‐MSCs	Chile (South America)
NCT02745379	Phase 1 Unknown status (January 2016)	20	Alveolar bone loss	BFPSCs	Iran (Asia)
NCT04998058	Phase 1 Phase 2 Not yet recruiting. (December 2025)	20	Alveolar bone loss, Alveolar bone atrophy	Autogenous MSCs	Brazil (South America)
NCT02745366	Phase 1 Unknown status (January 2016)	20	Alveolar bone loss, Atrophy.	BFPSCs	Iran (Asia)
NCT02209311	Phase 1 Phase 2 Unknown status (September 2014)	12	Alveolar bone loss, Alveolar bone atrophy.	Oral MSCs	Russian (Europe and Asia)
NCT04297813	Phase 3 Recruiting (March 2020)	48	Alveolar bone atrophy	Autogenous MSCs	Denmark, France, Norway, Spain (Europe)
NCT03638154	Not Applicable Completed (March 2016)	20	Periodontal intrabony defect, Periodontitis	GMSCs	NA

*Note:* Main observations: (1) 70.6% (12/17) trials were Phase 1/2; (2) geographic distribution showed 47.1% (8/17) in Asia (including transcontinental Turkey/Russia), 35.3% (6/17) in Europe, and 23.5% (4/17) in the Americas (1 North, 3 South); and (3) dental‐origin MSCs (DPSCs/PDLSCs) comprised 29.4% (5/17) of therapeutic approaches.

Abbreviation: BFPSCs, buccal fat pad derived mesenchymal stem cells.

## 5 Limitations and Future Perspectives

While MSCs hold significant promise for periodontal regeneration, several challenges must be addressed before their widespread clinical translation. Key limitations include variability in cell sources, mechanistic uncertainties, safety concerns, and socioeconomic barriers.

### 5.1 Cellular Source Considerations and Standardization Challenges

The therapeutic efficacy of MSCs is fundamentally influenced by their tissue of origin, with BM‐MSCs, AD‐MSCs, and DPSCs representing the most clinically relevant sources. These distinct MSC populations exhibit marked variations in their proliferative capacity, differentiation potential, and immunomodulatory properties. BM‐MSCs demonstrate superior osteogenic capacity compared to other MSC sources, as indicated by their enhanced mineralization potential even without osteogenic induction (Hoseini and Montazeri 2025). This intrinsic property makes them particularly suitable for periodontal bone regeneration. In contrast, AD‐MSCs offer practical advantages in clinical settings due to their minimally invasive harvesting procedure (typically via liposuction) and superior expansion capacity in vitro, as evidenced by their shorter population doubling time and maintenance of stemness properties through higher passages (Mazini et al. 2019). Although slightly less potent in osteogenic differentiation than BM‐MSCs, their differentiation potential can be effectively enhanced through specific induction protocols. DPSCs exhibit inherent advantages for periodontal regeneration due to their odontogenic lineage commitment and neural crest‐derived properties. As evidenced by Nakashima et al. (Aydin and Şahin 2019), these cells exhibit superior tissue‐specific differentiation potential and secrete unique regenerative factors that orchestrate periodontal wound healing. This inherent heterogeneity presents significant standardization challenges in MSC‐based therapies, as variations in isolation protocols, culture conditions, and characterization methods can substantially impact cell phenotype, functionality, and ultimately clinical outcomes (Mushahary et al. 2018).

As Rad et al.'s systematic review (2022) demonstrates, significant variations exist across PDLSC studies with regard to cell characterization, administration methods, and outcome measures. For the purpose of cell characterization, the majority of studies utilize conventional mesenchymal markers, such as CD73/CD90/CD105, while certain studies incorporate dental‐specific markers, including STRO‐1 and CD146. A number of studies employed immunocytochemistry (ICC) as the sole method for surface marker analysis, without utilizing flow cytometry. Furthermore, certain research identified cell characteristics solely through histological evaluation and differentiation testing, with a lack of flow cytometry verification. Functional assays such as clonogenicity (CFU) and differentiation potential (osteogenic/adipogenic) vary in induction duration (8–14 days) and assessment methods (Alizarin Red vs. von Kossa staining). Administration methods vary across studies, with the majority employing ex vivo/in vivo transplantation through subcutaneous implantation using fibrin gel or HA/TCP ceramic carriers. In terms of outcome measures, Rad et al. compared the effects of different isolation methods on cellular characteristics (such as mineralization and gene expression) by pooling data from CFU, cell proliferation assays, and gene expression studies across various studies. Overall, the differences between research primarily stemmed from variations in isolation methods, cellular subpopulation heterogeneity, and experimental design (Rad et al. 2022).

Research methods for cell isolation, expansion, and quality assessment also exhibit variations. The most typical cell separation methods primarily include enzymatic digestion and the explant method. Enzymatic digestion uses enzymes such as collagenase or trypsin to dissociate tissue into a single‐cell suspension, typically yielding high cell yields but causing some damage to cells, which can affect their activity and proliferative capacity. The explant method, in contrast, utilizes the action of placing tissue pieces in culture medium and relying on cells spontaneously migrating out for separation; although cell proliferation is slower, this method preserves better cell morphology and functional characteristics. Additionally, diverse separation methods have diverse effects on stem cell subpopulations. For example, enzymatic digestion may lead to the preferential growth of cells with high proliferative activity, while the explant method better preserves cells' multipotent differentiation potential. Moreover, the composition of the culture medium and the addition of the type and concentration of serum and the use of cytokines also significantly influence MSCs proliferation and differentiation capacity. Some studies have also proposed the use of serum‐free media to reduce serum variability and improve the consistency of experiments (Mushahary et al. 2018). Therefore, standardized separation and culture methods are lacking for a detailed assessment of cell quality. The minimum standards proposed by the International Society for Cell Therapy (ISCT), such as self‐renewal capacity, differentiation potential, and expression of surface markers, are widely recognized assessment methods, while additional functional tests can help supplement and improve the quality evaluation system.

In summary, while the variables in the studies exerted varying degrees of influence on the final outcomes, these variables provide important evidence for a deeper understanding of the potential of MSCs, while also laying a data foundation for the standardization of future research and clinical applications. The differences in research protocols also highlight the urgent need for standardized methods, which are crucial for ensuring the comparability of results across studies. Additionally, implementing a unified long‐term safety assessment system across different studies will significantly promote the translation of periodontal therapy based on MSCs into clinical applications. These standardization requirements align with and are further specified by current regulatory frameworks for cell‐based therapies.

Current Good Manufacturing Practice (cGMP) standards mandate rigorous quality control measures, including assessments of cell viability, purity, differentiation capacity, and microbiological safety (Gavin 2025). Regulatory bodies such as the Food and Drug Administration (FDA) and European Medicines Agency (EMA) have established stringent requirements for cell therapy products, encompassing production protocols, cryopreservation methods, and distribution logistics, thereby adding layers of complexity to clinical translation.

### 5.2 Mechanistic Gaps and Long‐Term Efficacy

While MSCs are known to exert therapeutic effects through multiple mechanisms including paracrine signaling (Chang et al. 2021) (e.g., secretion of VEGF, FGF, TGF‐β), extracellular vesicle‐mediated communication, and immunomodulation through macrophage polarization (Qiu et al. 2019), the precise molecular pathways underlying these effects in periodontal regeneration remain incompletely understood. Specifically, critical knowledge gaps exist regarding how MSC‐secreted factors coordinate the complex processes of inflammation resolution, cell proliferation, and tissue remodeling in the periodontal niche. The dynamic interaction between transplanted MSCs, host immune cells, and periodontal pathogens requires particular investigation to optimize therapeutic protocols. Furthermore, current evidence suggests transplanted MSCs may have limited survival in periodontal tissues (Lin et al. 2021), highlighting the need to develop advanced delivery systems like biomaterial scaffolds or hydrogels that could prolong cellular retention and enhance regenerative potential. A deeper understanding of MSC secretome composition and its temporal regulation during periodontal healing will be crucial for developing more effective cell therapies. Future studies should focus on elucidating the specific signaling pathways governing MSC‐host cell interactions, while longitudinal assessments are needed to determine the durability of MSC‐mediated tissue regeneration. These mechanistic insights will be essential for translating MSC therapies into clinically robust periodontal treatments.

### 5.3 Safety and Immunological Considerations

The clinical translation of MSCs‐based therapies necessitates careful evaluation of safety profiles. In this context, two primary safety concerns emerge prominently: tumorigenicity and immunogenicity. Regarding tumorigenicity, prolonged in vitro expansion during cell passaging may lead to genetic instability, potentially increasing risks of malignant transformation (Neri 2019). While such occurrences remain rare, regulatory agencies recommend strict limits on passage numbers and mandatory genomic stability testing prior to clinical application. The second major concern, immunogenicity, presents a complex paradox. Although MSCs possess inherent immunomodulatory properties through secreted factors like IDO and PGE2, allogeneic transplantation in inflammatory environments may trigger unexpected immune responses. This dual nature of MSCs immunomodulation requires particular attention in periodontitis treatment, where the existing inflammatory microenvironment could potentially alter therapeutic outcomes (Pasquinelli and Ciavarella 2019). To address these challenges, a comprehensive strategy should be implemented, including: (1) rigorous preclinical trials in relevant animal models; (2) standardized tumorigenicity analyses; (3) consideration of genetic engineering approaches to enhance safety profiles; and (4) in clinical trials, careful immune monitoring to evaluate both short‐term reactions and long‐term immunological consequences.

In recent years, gene engineering methods have shown significant promise in improving the safety of MSCs. Studies have demonstrated that suicide gene therapy using stem cells exhibits significant efficacy against various cancers, including non‐small cell lung cancer (NSCLC), breast cancer, and colorectal cancer. Oishi et al. developed an innovative gene engineering strategy using human deciduous tooth‐derived mesenchymal stem cells (SHED) as carriers for suicide gene therapy to combat brain metastasis in non‐small cell lung cancer. The researchers transduced the modified herpes simplex virus thymidine kinase (HSV‐TK) gene into SHED and enhanced the cells' antitumor effects through the HSV‐TK/GCV system. Notably, Oishi et al. introduced an A168H mutation into the HSV‐TK gene, which significantly reduced toxicity to stem cells while maintaining high transduction efficiency and GCV metabolic efficacy. Specifically, the research team optimized the transduction conditions of HSV‐TK (including transduction concentration MOI), enabling transduced SHED cells to effectively exert antitumor effects while maintaining cellular viability. Additionally, by validating the migration capacity and bystander effect of SHED cells, the researchers confirmed that this suicide gene therapy method can kill surrounding tumor cells through “bystander effect”, thereby enhancing therapeutic efficacy. This strategy can serve as a genetic engineering approach to enhance MSC safety, as mutations reduce toxicity to MSCs themselves while still effectively performing therapeutic functions. These evidence‐based genetic modifications not only demonstrate how engineering methods can simultaneously enhance the safety and therapeutic potential of MSC‐based therapies but also provide a framework for future clinical translation (Oishi et al. 2022).

### 5.4 Ethical and Economic Considerations

The widespread clinical implementation of MSC therapies encounters significant ethical and economic hurdles. The substantial costs associated with cell isolation, expansion, and quality control constitute major barriers to accessibility (Pereira Chilima et al. 2018). These expenses arise from the necessity for specialized equipment, high‐grade culture media, and comprehensive quality assurance measures. Ethical considerations predominantly focus on cell sourcing and donor consent (Volarevic et al. 2018). While certain MSC sources, such as adipose tissue, allow for relatively straightforward procurement, others, like UC‐MSCs, present more complex ethical dilemmas concerning informed consent and donor rights.

Additionally, in clinical practice, MSCs from different sources pose certain donor‐related risks, particularly issues such as viral contamination and immune reactions. First, MSCs may carry viral genetic material from donors, such as human immunodeficiency virus (HIV) or other types of viruses, which requires strict control and testing during cell culture and storage. Moreover, MSCs are prone to morphological changes, proliferation inhibition, or chromosomal abnormalities during in vitro culture, especially cells cultured for extended periods (over nine generations), which may lead to adverse outcomes. Furthermore, major adverse reactions associated with MSC therapy include thromboembolism and fibrosis, particularly with poorly differentiated cells or MSCs from elderly donors, which may increase the risk of immunosuppression and thrombosis. Such reactions are often related to individual genetic characteristics and differences in cell origin, and patients must be made aware of these potential risks in the informed consent form. Consequently, prior to clinical application, rigorous inspection of MSC sources, processing, and quality is essential, including PCR testing to rule out infections such as mycoplasma and herpesviruses, to ensure cell purity and safety. To avoid unnecessary complications, it is recommended to use minimally manipulated cells (e.g., MSCs after cryopreservation) to reduce the risk of variation (Baranovskii et al. 2022).

Building on this foundation, three key strategies are emerging to further enhance safety and ethical compliance: (1) adoption of alternative cell sources like non‐cultured, directly cryopreserved stromal cells which minimize genetic instability risks associated with long‐term culture, (2) utilization of MSC‐derived exosomes—particularly those from hypoxia‐preconditioned cultures—that retain therapeutic potential while eliminating risks of uncontrolled differentiation, and (3) establishment of centralized banking systems with standardized processing protocols. These approaches align with evolving global regulations favoring minimally manipulated cellular products, though they require careful balancing of innovation with responsible translation. While exosome‐based therapies and fresh‐frozen cells demonstrate improved safety profiles by avoiding chromosomal abnormalities and differentiation‐related complications, their implementation must be coupled with robust consent frameworks and quality controls to ensure both treatment accessibility and ethical compliance.

### 5.5 Clinical Translation Challenges

Although MSC‐based therapies demonstrate considerable potential for periodontal regeneration, their clinical translation—particularly for PDLSCs—is impeded by a multifaceted combination of biological and technical barriers. It is evident that PDLSCs manifest inherent constraints, encompassing age‐dependent functional deterioration (i.e., diminished proliferation, migration, and differentiation potential within mature tissues) and variable responses to biomaterial/cytokine stimulation. These fundamental constraints result in ambiguous preclinical outcomes, with many trials stagnating at the preclinical stage. In addition, the clinical efficacy of autologous PDLSC transplantation has been shown to be inconsistent; thus, highlighting the limitations of cell‐based strategies when used in isolation. These issues are further compounded by the presence of microenvironmental regulation challenges, where immune factors, metal ions, and bioactive cues require precise spatiotemporal control. This represents a significant hurdle that has yet to be overcome despite advances in scaffold and cell‐sheet technologies.

In order to maximize the clinical potential of PDLSCs, a coordinated framework must be established to address the following points. The first element is the standardization of cell sourcing protocols, with consideration given to donor age and tissue status. The second element is the development of biomaterials that mimic the properties of the niche, with the aim of balancing scalability with cost‐effectiveness. The third element is multifactorial precision control systems, which are used to reliably activate PDLSCs multipotency. These innovations must be included in well‐designed multicenter RCTs that evaluate long‐term safety, functional outcomes, and ethical‐economic feasibility. Only through this comprehensive approach, comprehensive immunomodulation, advanced delivery systems, and mechanism knowledge can the ongoing gap between in vitro results and consistent clinical translation be bridged.

## 6 Conclusion

Current perspectives on MSCs underscore their transformative potential as a treatment for periodontal diseases, offering regenerative solutions that extend beyond conventional therapies. Preclinical evidence highlights their capacity for immunomodulation and tissue repair. However, the clinical translation of these findings necessitates addressing challenges related to standardization, safety, and delivery. Moving forward, conducting rigorous clinical trials with consistent outcome measures and long‐term evaluations will be critical to validate MSCs‐based therapies. As advancements in this field progress, MSCs may establish themselves as a valuable therapeutic option, particularly for periodontal cases. The evolving understanding of MSCs applications promises to shape the future of periodontal regenerative medicine, bridging the gap between experimental promise and clinical reality.

## Author Contributions

Conceptualization: Jincheng Zeng, Mingdeng Rong; Writing – original draft preparation: Shuyu Cai and Cong Li; Writing – review and editing: Biaowen Wei, Wanxiang Ye, and Ziyu Ye; Funding acquisition: Shuyu Cai, Mingdeng Rong, Qin Mao, and Wanxiang Ye. All authors have read and agreed to the published version of the manuscript.

## Ethics Statement

The authors have nothing to report.

## Consent

The authors have nothing to report.

## Data Availability

The data that support the findings of this study are available in Clinicaltrials at https://clinicaltrials.gov.
